# Machine learning improves prediction of pulmonary thromboembolism and reduces unnecessary computed tomography scans in the emergency department

**DOI:** 10.1038/s41598-025-34952-x

**Published:** 2026-01-09

**Authors:** Sung Hyun Yoon, Cheolho Kwon, Yeongho Choi, Hyung-Jun Kim, Jihang Kim, Young Hoon Kim

**Affiliations:** 1 Department of Radiology, Seoul National University Bundang Hospital, Seongnam, Republic of Korea; 2 Department of Emergency Medicine, Seoul National University Bundang Hospital, Seongnam, Republic of Korea; 3 Division of Pulmonary and Critical Care Medicine, Department of Internal Medicine, Seoul National University Bundang Hospital, Seongnam, Republic of Korea; 4 Department of Internal Medicine, Seoul National University College of Medicine, Seoul, Republic of Korea; 5 Department of Radiology, Seoul National University College of Medicine, Seoul, Republic of Korea

**Keywords:** Pulmonary embolism, Computed tomography pulmonary angiography, Clinical prediction rules, Supervised machine learning, Health care, Medical research

## Abstract

The diagnosis of pulmonary thromboembolism (PTE) remains challenging due to its nonspecific clinical signs and symptoms. This study aimed to develop a machine learning (ML) model to predict PTE in emergency department patients. We retrospectively analyzed 2,525 emergency department patients suspected of PTE who underwent computed tomography pulmonary angiography (CTPA) within 7 days after elevated D-dimer levels (≥ 0.5 µg/ml) at a tertiary hospital, between January 2012 and December 2021. Clinical and laboratory data were split into training (*n* = 2025) and test (*n* = 500) sets. Six ML models—XGBoost, random forest, logistic regression, elastic net regression, support vector machine, and feed-forward neural network—were compared with the revised Geneva score using the area under the receiver operating characteristic curve (AUC). Variable importance was assessed using permutation methods. Of the 2,525 patients, 573 (22.7%) were diagnosed with PTE. XGBoost achieved the highest AUC of 0.814 (95% confidence interval [CI]: 0.759–0.862). All ML models outperformed the revised Geneva score, which had an AUC of 0.622 (95% CI: 0.563–0.675). D-dimer and activated partial thromboplastin time were the most important predictors across all ML models. At sensitivities of 100%, 95%, and 90%, the XGBoost model could reduce the number of CTPA scans by 3.0%, 14.8%, and 33.2%, respectively (all *p* < 0.001). These findings suggest that ML models, particularly XGBoost, can improve PTE risk prediction compared to the revised Geneva score and may help reduce unnecessary CTPA imaging in the emergency department.

**Supplementary Information:**

The online version contains supplementary material available at 10.1038/s41598-025-34952-x.

## Introduction

Pulmonary thromboembolism (PTE) represents a significant clinical challenge in emergency medicine and is characterized by the sudden obstruction of the pulmonary arteries by thrombi, typically caused by embolization of thrombi originating from deep vein thrombosis (DVT). PTE poses a substantial burden on healthcare systems worldwide with an annual incidence of approximately 1 in 1000 persons; PTE is the third most common cardiovascular syndrome globally, following myocardial infarction and stroke^1,2^. Prompt recognition and optimal management can reduce mortality and morbidity in patients with PTE^3^. Delayed diagnosis and inadequate treatment can lead to adverse outcomes, including sudden death, recurrent thromboembolic events, and chronic pulmonary hypertension^4^.

However, the diagnosis of PTE remains challenging because of its nonspecific clinical signs and symptoms, such as dyspnea, chest pain, and tachycardia, which can mimic various other conditions. While various predisposing factors for PTE are recognized, approximately 40% of patients with PTE do not exhibit any known predisposing factors^5^. Clinical prediction rules, such as the Wells score and Geneva score, are commonly used alongside D-dimer testing to assess the likelihood of PTE and guide further diagnostic testing^3^. These rules aim to rule out PTE by identifying low-risk patients. However, their performance in predicting PTE risk is limited, as many high-risk patients are not diagnosed with PTE^6–9^. Elevated D-dimer levels also have a low positive-predictive value for confirming PTE. In addition, D-dimer is more frequently elevated in older patients^10,11^, patients with cancer^12,13^, hospitalized patients^14^, and cases of severe infection or inflammatory disease, leading to a high number of false-positives in these groups. Given the limited discriminative performance of clinical prediction rules and D-dimer testing, diagnostic algorithms that rely on them are associated with a low diagnostic yield for computed tomography pulmonary angiography (CTPA). Reported positivity rates are 11.2% overall and up to 33.1% in patients with high pretest probability^15,16^. These yields suggest that the current diagnostic algorithms may increase the number of unnecessary CTPA examinations, resulting in undue radiation exposure and higher healthcare costs.

These issues illustrate recurring challenges in decision-making across acute and critical-care settings. Recent studies have begun to address these limitations through machine learning (ML) approaches. By integrating diverse clinical variables and identifying patterns often overlooked in complex environments, ML models have demonstrated superior predictive performance over conventional risk scores and clinical judgment^17–19^. ML models have also shown promising performance in predicting the risk of venous thromboembolism (VTE), including PTE and DVT^20–24^. However, these previous ML models were developed primarily in inpatient settings, and studies on emergency department patients remain scarce. Many of these models relied on International Classification of Diseases (ICD) codes or antithrombotic treatment history for the confirmation of PTE, rather than explicit imaging-based verification. Such non-standard confirmation limits clinical applicability and underscores the need for models built on reliable datasets verified by the diagnostic method of choice. Therefore, in this study, we aimed to develop an ML-based prediction model for PTE in the emergency department using CTPA-confirmed diagnoses, with the goal of reducing unnecessary CTPA scans.

## Methods

### Setting and selection of participants

This retrospective study was approved by the Institutional Review Board of Seoul National University Bundang Hospital (IRB No. B-2307-840-108). The requirement for obtaining patient informed consent was waived by the Institutional Review Board of Seoul National University Bundang Hospital because of the retrospective nature of the study. This study was reported in accordance with the Transparent Reporting of a Multivariable Prediction Model for Individual Prognosis or Diagnosis with Artificial Intelligence (TRIPOD + AI) guidelines^25^.

**Fig. 1:**
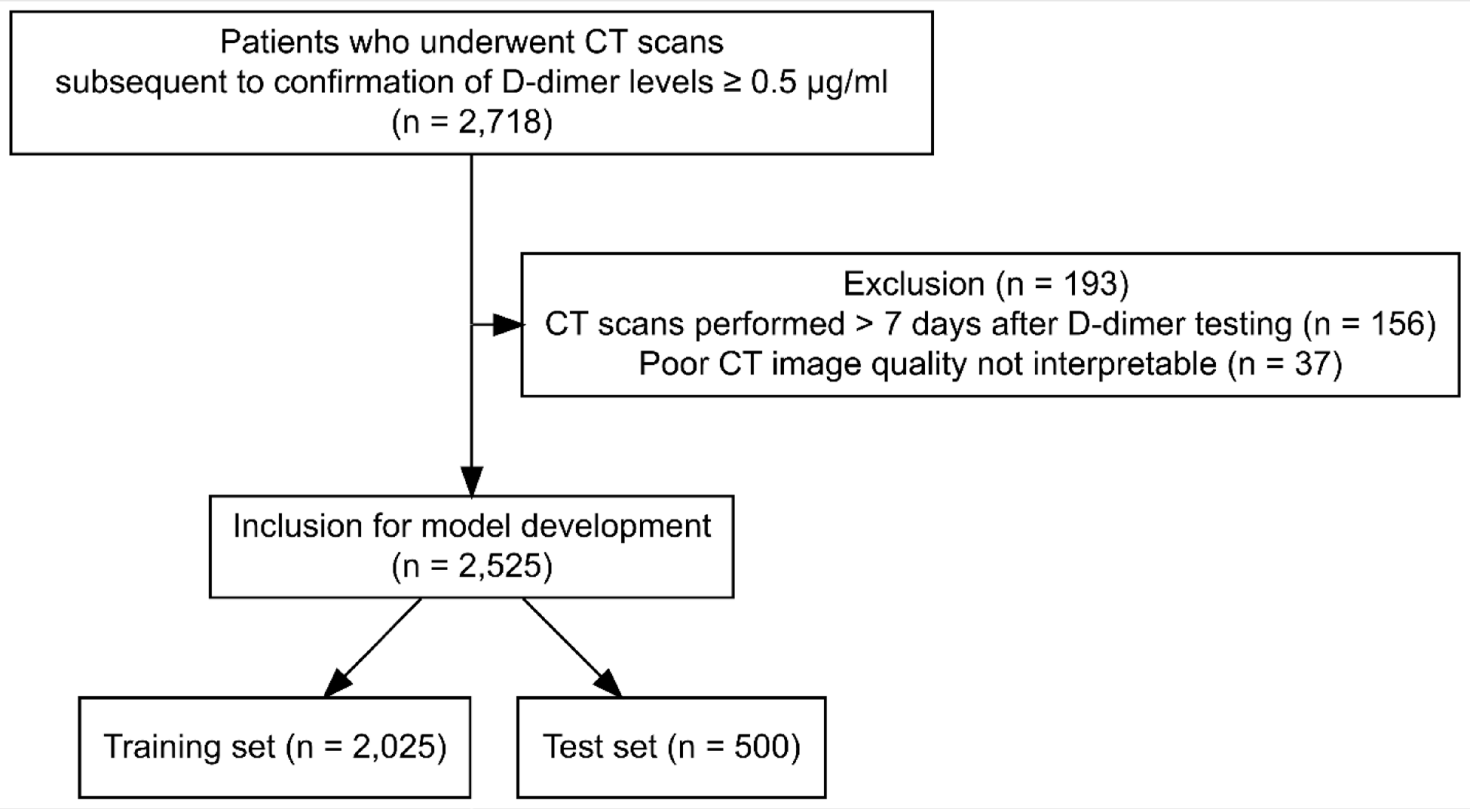
Flow chart diagram for this study.

This study was conducted at a tertiary academic hospital (Seoul National University Bundang Hospital) with 60,000–90,000 emergency department visits annually. We retrospectively identified consecutive patients suspected of having PTE who underwent computed tomography (CT) scans subsequent to the confirmation of D-dimer levels ≥ 0.5 µg/ml ^26^ in the emergency department, from January 1, 2012, to December 31, 2021. Only the initial visit to the emergency department was considered for each patient. Patients were excluded if CT was performed > 7 days after D-dimer testing or if the CT was non-diagnostic due to poor enhancement of the main pulmonary artery (< 250 HU) or severe motion artifact (Fig. 1). In total, 2,525 patients were included in this study.

Although there is no universally accepted method for estimating the required sample size in ML models, we adopted Riley’s approach^27^ for calculating the sample size in the development of our clinical prediction models. To ensure accurate predictions and minimize the risk of overfitting, the sample size calculation should account for the number of events, total number of participants, proportion of the outcome, and expected predictive performance of the model^27^. Based on local clinical experience and a previous study^28^, we estimated the prevalence of PTE in emergency department patients with elevated D-dimer levels to be at least 20%. With an anticipated model performance (AUC) of 0.82^20,29^ and 41 predictors, the minimum required sample size for this study was calculated to be 1566 participants in the training cohort, confirming that our sample size is sufficient for model development. The *pmsampsize* package in R was used to perform the sample size calculation according to Riley’s method.

### Outcome

The presence of PTE was confirmed using CT. In our institution, CT pulmonary angiography employed the bolus-tracking method, with image acquisition triggered when the attenuation of the proximal pulmonary artery reached a threshold of 100 Hounsfield units (HU). Images were acquired using various CT scanners (Brilliance CT64, Brilliance iCT256, and IQon Spectral CT by Philips Healthcare, Best, The Netherlands, and SOMATOM definition edge, SOMATOM X.cite, and SOMATOM Force by Siemens Healthineers, Erlangen, Germany), including image reconstruction with a 1-mm slice thickness for pulmonary angiography. Intravenous contrast medium (Omnipaque 350, GE Healthcare, Chicago, IL, USA; Iomeron 350, BRACCO, Milan, Italy; and Xenetix 350, GUERBET, Villepint, France) was administered at a volume (ml) equivalent to twice the patient’s body weight (kg).

All CT images had been originally interpreted by fellowship-trained chest radiologists. The radiology reports were manually analyzed and labeled as “PTE-positive” when PTE was present. In cases where the radiology reports indicated an ambiguous diagnosis of PTE, the corresponding CT images were reviewed by a fellowship-trained chest radiologist (S.H.Y., with 3 years of experience) to determine the diagnosis.

### Measurements

Laboratory blood test results, vital signs, and medical histories were obtained from electronic medical records. All data were automatically retrieved using a health information system. Medical histories and symptoms were further supplemented by manual review, conducted between August 1 and October 31, 2023. During this period, the reviewer was blinded to patient outcomes. Authors had access to information that could identify individual participants after data collection. Among the results obtained within 7 days before or after D-dimer testing, only the initial results and those obtained prior to the CT examination were included.

Variables included age, sex, pulse rate, respiratory rate, systolic blood pressure, diastolic blood pressure, body temperature, body mass index. Laboratory test variables encompassed levels of D-dimer, fibrinogen, activated partial thromboplastin time (aPTT), and prothrombin time (international normalized ratio); C-reactive protein, absolute neutrophil count (ANC), segmented neutrophil percentage, eosinophil percentage, lymphocyte percentage, and white blood cell count (WBC); troponin I and creatine kinase; and hemoglobin, hematocrit, red blood cell count, platelet count, albumin, protein, and cholesterol levels. Variables concerning medical history included hypertension, diabetes, history of any venous thromboembolism event (PTE or DVT), history of prior major surgery or immobilization (> 3 days) within 1 month, presence of ischemic heart disease, atrial fibrillation or atrial flutter, active cancer, undergoing chemotherapy, history of prior ischemic stroke, presence of autoimmune disease (antiphospholipid antibody syndrome, systemic lupus erythematosus, inflammatory bowel disease, paroxysmal nocturnal hemoglobinuria, Behçet disease), coagulopathy (hereditary thrombophilia, protein C deficiency, protein S deficiency, antithrombin III deficiency, factor V Leiden mutation, prothrombin 20210 G-to-A variation, hyperhomocysteinemia, dysfibrinogenemia, familial plasminogen deficiency). Symptoms included unilateral leg pain and hemoptysis.

### ML models

Seven ML algorithms were used for model development: boosted trees, random forest, logistic regression, elastic net regression, support vector machine (SVM) with linear kernel, SVM with radial kernel, and a feed-forward neural network. All ML models were developed using the open-source program R version 4.3.2 (R Foundation for Statistical Computing, Vienna, Austria).

Laboratory test variables with more than 16% missingness were excluded. Among the initial study population of 2525 patients, 1857 had complete data for the remaining variables. Missing continuous variables were then imputed using the k-nearest neighbors (kNN) imputation method (k = 10) implemented in the *VIM* package^30^ of R. To ensure data completeness in performance evaluation, the test set was sampled from patients with originally complete data. Of the 1,857 patients with initial complete data, 500 (20% of the total population) were randomly selected as the test dataset and the remaining 2025 patients were used for model training. The proportions of missing values for each laboratory variable are summarized in Supplementary Tables 1, and the resulting distribution of variables in the training and test sets is summarized in Supplementary Table 2. Pairwise correlations among all numeric and binary variables were examined using Spearman correlation coefficients in the training dataset. Binary variables were coded as 0/1, with 1 indicating the presence of the corresponding clinical condition (e.g., hypertension or diabetes). No variables were excluded on the basis of correlation, and no explicit outlier removal was performed. All input variables were normalized to have a standard deviation of one and a mean of zero using the *recipes* package^31^.

The logistic regression model used the *stats* package in R. Initially, univariate logistic regression analysis was conducted to identify significant variables. All variables with significance < 5% in the univariate analysis were included in the multiple logistic regression model, and backward elimination was used to determine the final variables. Other ML algorithms, including boosted trees, random forest, elastic net regression, SVM, and feed-forward neural network, used the *XGBoost*^32^, *ranger*^33^, *glmnet*^34^, *kernlab*^35^, and *nnet*^36^ packages under the *tidymodels* framework^37^ in R. The model hyperparameters were optimized using a five-fold cross-validated grid search. During each cross-validation iteration, four-fifths of the training set were randomly chosen for model training, whereas the remaining one-fifth served as the validation set. Subsequently, the results of five cross-validation iterations were aggregated to derive the overall model evaluation results. This process was repeated across a parameter grid. The initial search space was defined based on recommended defaults in prior literature^32–36^, and the grid was iteratively refined by monitoring cross-validated AUC, model stability, and indicators of potential overfitting (e.g., divergence between training and validation performance). The combination of hyperparameters that yielded the most stable and highest cross-validated performance was selected and incorporated into the final model, which was then trained using the entire training set, and evaluated using the test set. Specific hyperparameters were tuned for each model: nrounds, eta, max_depth, and colsample_bytree for boosted trees; mtry, num.trees, and min.node.size for random forest; lambda and alpha for elastic net regression; C for linear SVM; C and sigma for radial SVM; and size, maxit, and decay for the feed-forward neural network.

### Analysis

The performance of each model on the test dataset was assessed using the area under the receiver operating characteristic curve (AUC). The sensitivity, specificity, accuracy, and F1 score were calculated for the model operating points, with the sensitivity fixed at 0.9. We used bootstrapping with 1000 resamples to calculate the 95% confidence intervals (CIs) for the AUC, sensitivity, specificity, accuracy, and F1 score. To evaluate the quality of predicted probabilities, model calibration was assessed using both calibration plots and the Brier score. Performance of the revised Geneva score^38^ was assessed using the same metrics described above. Subsequent analyses were conducted using the model with the highest AUC. As a supplementary comparison, we conducted a decision-curve analysis. The threshold probability range was set to 0–30%, covering the probability level below which further testing is generally unnecessary (approximately 1.4%)^39^ and extending to the intermediate-risk range of the revised Geneva score (approximately 28%)^38^, where additional evaluation is often required. We also analyzed the relationship between the ML probability score and the incidence of PTE. In addition, we explored various thresholds to rule out PTE in the test dataset. Statistical analyses were performed using the open-source program R version 4.3.2 (R Foundation for Statistical Computing, Vienna, Austria).

We used a model-agnostic method known as permutation-based variable importance (PVI)^40^ to explain the model predictions. PVI measures the significance of a variable by calculating the increase in a model’s prediction error after permuting the variable. The underlying rationale is that permuting the values of an important variable in the training data degrades training performance because permuting the values of a variable disrupts the relationship between the predictor and outcome variables. PVI involves comparing the difference between the baseline performance measure (e.g., 1 - AUC) and the performance measure obtained after permuting the values of a particular variable in the training data. When a larger change in performance is observed, the variable is deemed more important. The PVI was calculated and visualized using the *DALEX*^41^ and *DALEXtra*^42^ packages in R. Permutations were conducted 30 times, each using 1,000 samples of data.

## Results

### Characteristics of study subjects

Patient characteristics are summarized in Table 1 and Supplementary Tables 1 and 2. Of 2525 patients included in this study, 573 (22.7%) developed PTE. In the training dataset (*n* = 2,025), 461 patients (22.8%) had PTE and in the test dataset (*n* = 500), 112 patients (22.8%) had PTE. In the training dataset, pairwise correlation analysis identified strong associations (|r| ≥ 0.7) only among a few physiologically linked variables such as hemoglobin–hematocrit and WBC–ANC. The full correlation matrix is provided in Supplementary Fig. S1.

**Table 1:** Characteristics of patients with and without PTE.

Characteristic	Overall (*n* = 2,525)	PTE Negative (*n* = 1,952)	PTE Positive (*n* = 573)	*p*-value
Sex				0.007
M	1,214.0 (48.1%)	967.0 (49.5%)	247.0 (43.1%)	
F	1,311.0 (51.9%)	985.0 (50.5%)	326.0 (56.9%)	
Age (years)				0.003
Mean ± SD	68.9 ± 16.2	69.4 ± 16.0	67.1 ± 16.7	
Median (IQR)	73.0 (60.0, 80.0)	73.5 (61.0, 81.0)	71.0 (57.0, 79.0)	
HTN	1,131.0 (44.8%)	884.0 (45.3%)	247.0 (43.1%)	0.364
DM	627.0 (24.8%)	507.0 (26.0%)	120.0 (20.9%)	0.015
Previous VTE	237.0 (9.4%)	140.0 (7.2%)	97.0 (16.9%)	< 0.001
Surgery or immobilization	438.0 (17.3%)	333.0 (17.1%)	105.0 (18.3%)	0.490
Ischemic heart disease	184.0 (7.3%)	159.0 (8.1%)	25.0 (4.4%)	0.002
Atrial fibrillation or atrial flutter	243.0 (9.6%)	204.0 (10.5%)	39.0 (6.8%)	0.010
Stroke	258.0 (10.2%)	210.0 (10.8%)	48.0 (8.4%)	0.100
Active cancer	725.0 (28.7%)	554.0 (28.4%)	171.0 (29.8%)	0.495
Chemotherapy	383.0 (15.2%)	299.0 (15.3%)	84.0 (14.7%)	0.741
Autoimmune disease	27.0 (1.1%)	23.0 (1.2%)	4.0 (0.7%)	0.487
Coagulopathy	11.0 (0.4%)	3.0 (0.2%)	8.0 (1.4%)	< 0.001
Anticoagulation	412.0 (16.3%)	320.0 (16.4%)	92.0 (16.1%)	0.898
Unilateral leg pain	316.0 (12.5%)	191.0 (9.8%)	125.0 (21.8%)	< 0.001
Hemoptysis	4.0 (0.2%)	4.0 (0.2%)	0.0 (0.0%)	0.580

Unless otherwise specified, data in parentheses represent percentages. P-values were calculated using Fisher’s exact test for dichotomous variables and Welch’s Two-Sample t-test for continuous variables.

HTN, hypertension; DM, diabetes; VTE, venous thromboembolism.

### Model performance

**Fig. 2:**
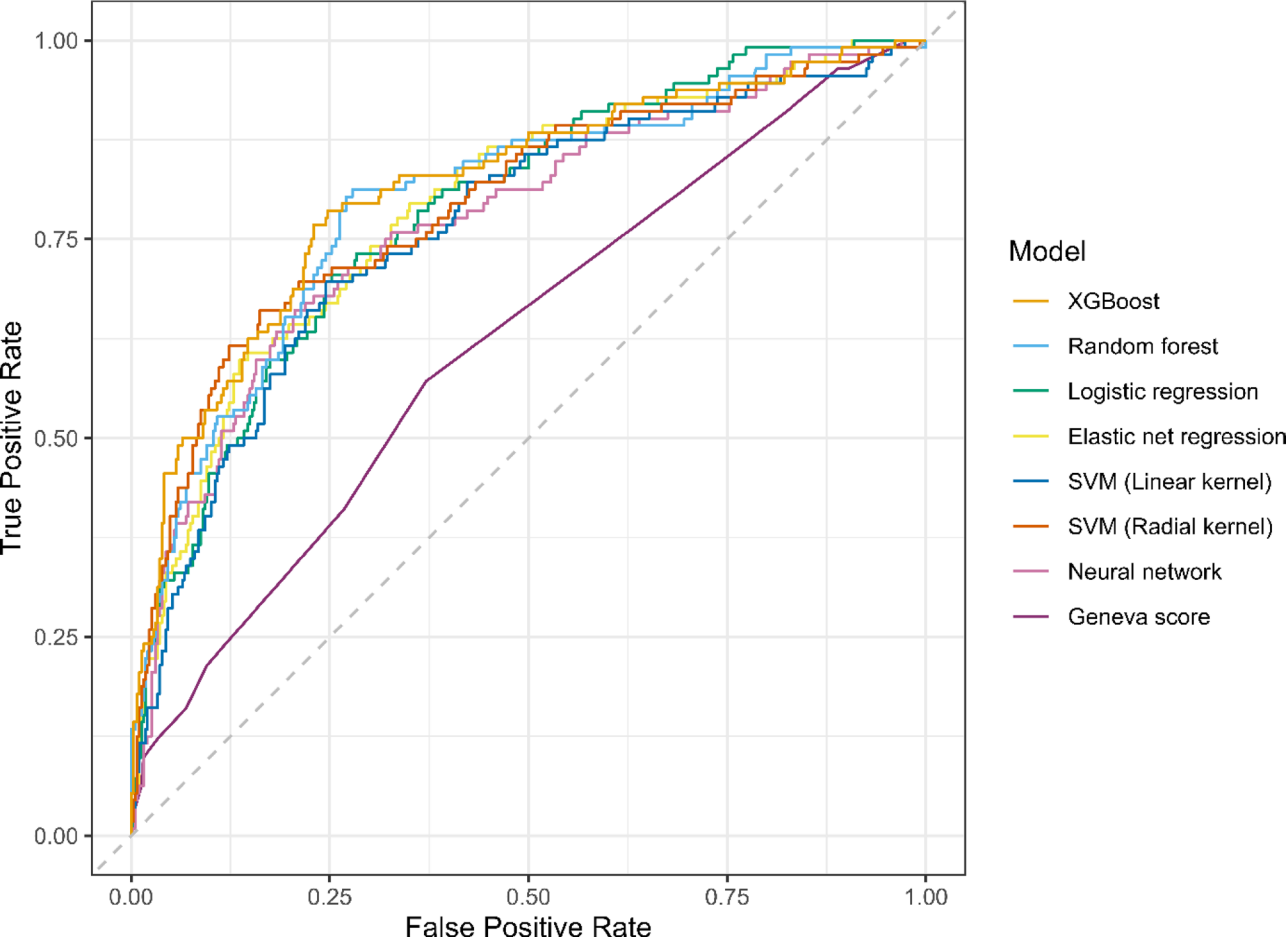
Receiver operating characteristic curves for machine learning models.

**Fig. 3:**
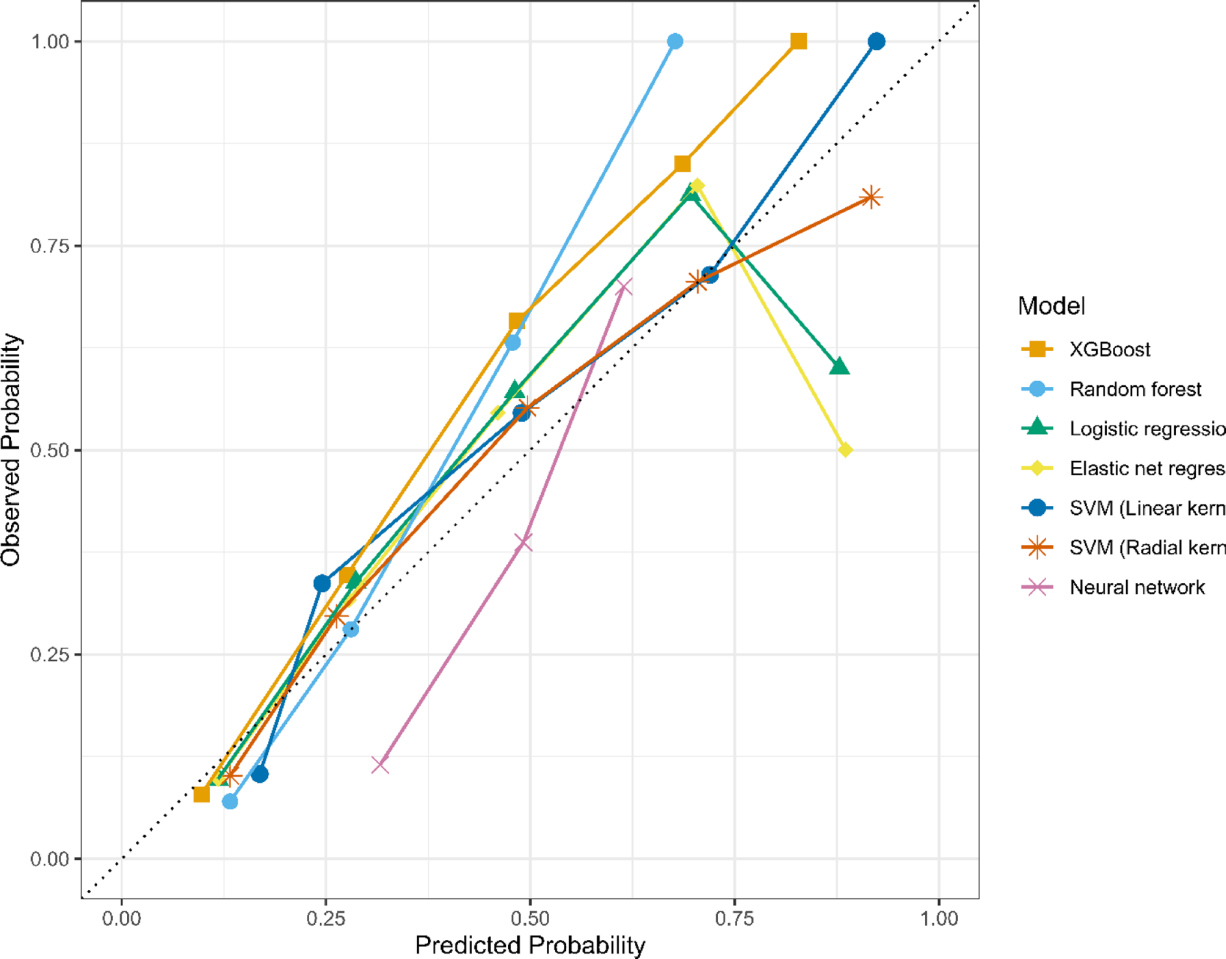
Calibration curves for machine learning models.

The performance metrics of the ML models are summarized in Table 2; Fig. 2. Among the ML models considered, the XGBoost model demonstrated the best performance with the AUC of 0.814 (95% CI: 0.759–0.862). The random forest model performed similarly but showed a slightly lower AUC at 0.801 (95% CI: 0.753–0.850). At a fixed sensitivity of 90% across the models, the logistic regression model obtained the best specificity and accuracy. The AUC of the revised Geneva score was 0.622 (95% CI: 0.563–0.675), which was substantially lower than the AUCs of all ML models. The specifications and hyperparameters of the ML models are listed in Supplementary Table 3. XGBoost and both SVM models showed favorable calibration (Fig. 3), and XGBoost achieved the lowest Brier score. Taken together with its superior discrimination, XGBoost was identified as the overall best model. Supplementary decision curve analysis indicated greater net benefit for the continuous XGBoost model than for the revised Geneva score–based thresholds within the prespecified threshold probability range (0–30%) (Supplementary Fig. S1).

**Table 2:** Performance metrics of machine-learning models at a fixed sensitivity of 90%.

Model	AUC	Sensitivity	Specificity	Accuracy	F1	Brier Score
XGBoost	0.814 (0.759–0.862)	0.902(0.847–0.954)	0.399(0.35–0.449)	0.512(0.47–0.55)	0.453(0.395–0.51)	0.129
Random Forest	0.801 (0.753–0.850)	0.902(0.844–0.959)	0.304(0.255–0.349)	0.438(0.394–0.482)	0.418(0.362–0.47)	0.138
Logistic regression	0.786 (0.740–0.831)	0.902(0.845–0.953)	0.443(0.393–0.492)	0.546(0.504–0.592)	0.471(0.412–0.529)	0.140
Elastic net regression	0.790 (0.740–0.839)	0.902(0.843–0.956)	0.402(0.349–0.452)	0.514(0.472–0.554)	0.454(0.396–0.506)	0.139
Linear support vector machine	0.764 (0.710–0.814)	0.902(0.842–0.954)	0.374(0.326–0.42)	0.492(0.45–0.534)	0.443(0.387–0.497)	0.153
Radial support vector machine	0.793 (0.741–0.843)	0.902(0.846–0.955)	0.394(0.345–0.445)	0.508(0.468–0.552)	0.451(0.393–0.504)	0.134
Feed-forward neural network	0.772 (0.720–0.823)	0.902(0.837–0.954)	0.361(0.313–0.406)	0.482(0.44–0.524)	0.438(0.381–0.491)	0.169
Revised Geneva score	0.622 (0.563–0.675)	0.911(0.854–0.958)	0.175(0.138–0.213)	0.34(0.298–0.382)	0.382(0.328–0.434)	-

Data in parentheses represent 95% confidence intervals.

**Fig. 4:**
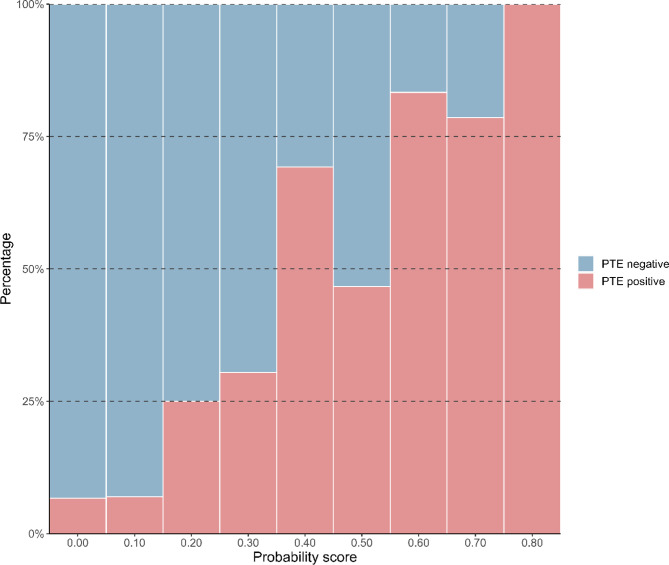
Proportional stacked histogram of PTE incidence by probability score.

Using XGBoost model, we observed a clear trend in which patients with higher probability scores were more likely to have PTE (Fig. 4). When we simulated the scenario in which patients were ruled out based on the probability score of the XGBoost model (Table 3), 15 of 500 (3%) patients could be safely ruled out for PTE, avoiding CTPA, without missing any PTE-positive cases. With the sensitivity thresholds set at 95.5% and 90.2%, CTPA was avoided in 74 of 500 (14.8%) and 166 of 500 (33.2%) patients, respectively.

**Table 3:** Results of screening scenarios using different probability score thresholds.

Sensitivity (%)	Probability score Threshold	Patients ruled out for PTE			*p*-value
		Total^*^	Normal	PTE	
100	0.036	15 (3%)	15 (100%)	0 (0%)	< 0.001
99.1	0.048	42 (8.4%)	41 (97.6%)	1 (2.4%)	< 0.001
98.2	0.049	44 (8.8%)	42 (95.5%)	2 (4.5%)	< 0.001
97.3	0.058	68 (13.6%)	65 (95.6%)	3 (4.4%)	< 0.001
96.4	0.058	70 (14%)	66 (94.3%)	4 (5.7%)	< 0.001
95.5	0.059	74 (14.8%)	69 (93.2%)	5 (6.8%)	< 0.001
94.6	0.071	107 (21.4%)	101 (94.4%)	6 (5.6%)	< 0.001
93.8	0.079	129 (25.8%)	122 (94.6%)	7 (5.4%)	< 0.001
92	0.093	161 (32.2%)	152 (94.4%)	9 (5.6%)	< 0.001
91.1	0.094	163 (32.6%)	153 (93.9%)	10 (6.1%)	< 0.001
90.2	0.095	166 (33.2%)	155 (93.4%)	11 (6.6%)	< 0.001

*Data in parentheses represent the percentage based on the entire study population. P-values were calculated using the McNemar test to compare the number of CTPA scans requested before and after the scenario simulation.

### Model interpretation

**Fig. 5:**
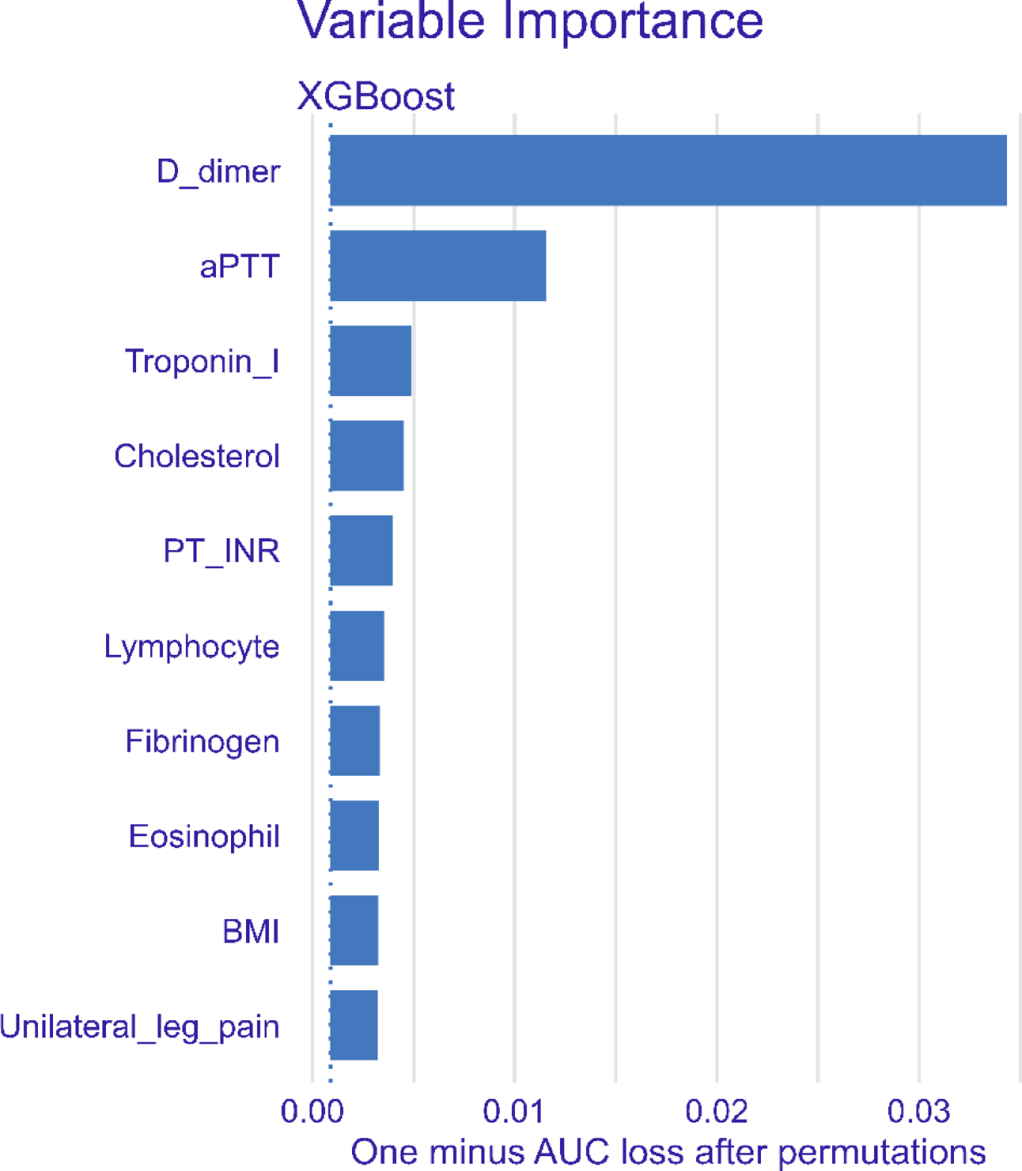
Variable importance plot of the XGBoost.

The variable importance plots of the XGBoost model were generated using the PVI and are presented in Fig. 5. The variable importance plots for the other models are presented in Supplementary Fig. S3. The top 10 ranked variables showed significant overlap across all ML models (Table 4). Notably, D-dimer level and aPTT consistently emerged as the top predictors across all ML models.

**Table 4:** Rankings of variable importance in machine-learning models.

Rank	Boosted tree (XGBoost)	Random Forest	Logistic regression	Elastic net regression	SVM (Linear kernel)	SVM (Radial kernel)	Feed-forward neural network
1	D_dimer	D_dimer	D_dimer	D_dimer	aPTT	aPTT	D_dimer
2	aPTT	aPTT	aPTT	aPTT	D_dimer	D_dimer	aPTT
3	Troponin_I	PT_INR	Unilateral_leg_pain	Unilateral_leg_pain	Unilateral_leg_pain	Fibrinogen	PLT
4	Cholesterol	Unilateral_leg_pain	ANC	PLT	Fibrinogen	Lymphocyte	Unilateral_leg_pain
5	PT_INR	Lymphocyte	Lymphocyte	Lymphocyte	PLT	Unilateral_leg_pain	Fibrinogen
6	Lymphocyte	Cholesterol	RBC	Fibrinogen	CRP	SBP	Prev_PTE_DVT
7	Fibrinogen	Eosinophil	Cholesterol	SBP	PT_INR	BMI	Afib_Af
8	Eosinophil	Troponin_I	Prev_PTE_DVT	Prev_PTE_DVT	Prev_PTE_DVT	PLT	Protein
9	BMI	BMI	BMI	RBC	Lymphocyte	Cholesterol	Anticoagulation
10	Unilateral_leg_pain	Prev_PTE_DVT	Fibrinogen	Cholesterol	Coagulopathy	Sex	RR

aPTT, activated partial thromboplastin time; PT_INR, prothrombin time (international normalized ratio); PLT, platelet count; ANC, absolute neutrophil count; RBC, red blood cell count; CRP, C-reactive protein; SBP, systolic blood pressure; Prev_PTE_DVT, previous pulmonary thromboembolism/deep vein thrombosis; BMI, body mass index; Afib_Af, atrial fibrillation; RR, respiratory rate.

## Discussion

In this study, we developed ML-based PTE risk prediction models and compared their performances in terms of the AUCs. All ML-based models surpassed the revised Geneva score, achieving AUCs over 0.76. Particularly, the XGBoost model exhibited the highest discriminative power with an AUC of 0.814 (95% CI: 0.759–0.862). The reliability of its probability estimates was supported by the lowest Brier score and a proportional increase in PTE incidence at higher predicted probabilities. Studies in other acute-care settings have shown that ML models provide more precise risk stratification than conventional scores^17,19^. Our findings demonstrate a similar advantage in the evaluation of suspected PTE, supporting the clinical relevance of ML-based prediction. Given that the second-best-performing model was random forest, ensemble methods, such as XGBoost and random forest, appear to outperform non-ensemble models. This pattern aligns with previous studies reporting that ensemble-based models yield superior discriminative performance in acute and critical care prediction tasks^18,43^. Ensemble methods offer several advantages, including the ability to aggregate predictions from multiple base models, balance the bias-variance tradeoff, increase model robustness to noise, and capture nonlinear relationships in data. These advantages likely play a pivotal role in uncovering the complex patterns associated with thromboembolic risk.

Based on our study, the ML-based model has the potential to reduce unnecessary CTPA scans in the emergency department. At a theoretical sensitivity of 100%, XGBoost was able to rule out only a small fraction of PTE-negative cases. However, when applying clinically reasonable operating points, such as a 95% sensitivity threshold, the model could achieve a modest but potentially meaningful reduction in unnecessary CTPA scans. This may not only reduce radiation exposure but also lower the risk of contrast-related adverse effects, such as nephropathy and anaphylaxis, thereby enhancing patient safety. To support clinical integration, the model can be embedded into electronic medical record systems to automatically generate a probability score from clinical and laboratory data. When the score falls below a predefined threshold, clinicians may consider deferring CTPA. Additionally, reducing redundant CTPA scans may help lower healthcare costs and alleviate emergency department congestion. Prospective and external validation of the threshold is essential to confirm its safety and generalizability across different clinical settings.

To evaluate the contribution of each variable to the model predictions, we used the PVI. Among the top 10 ranked variables, D-dimer and aPTT consistently emerged as dominant predictors across all ML models, significantly surpassing the contributions of other variables. The measurement of plasma D-dimer levels has long been recognized as a useful diagnostic tool for VTE^14,44^. The aPTT represents the activity of the coagulation system, which is closely related to thrombosis. Previous studies reported that shortened aPTT is associated with an increased risk of VTE^45,46^. In our study, however, patients with PTE exhibited slightly increased aPTT despite elevated D-dimer levels. Considering that a substantial proportion of our patients had cancer (28.7%) or were receiving anticoagulation therapy (16.3%), these factors might have inconsistently affected the aPTT levels in patients with and without PTE. This could result in a relative prolongation of aPTT in patients with PTE. The ML models utilized other PTE risk factors, such as history of VTE, and variables related to the coagulation system, such as fibrinogen level, PT INR, and platelet count.

The prediction of PTE risk is crucial for prevention and treatment. Various clinical prediction rules have been developed to stratify the risk of PTE. Several studies have evaluated the performance of the clinical prediction rules. In a prospective validation study evaluated the performance of five clinical prediction rules (The original Wells, modified Wells, simplified Wells, revised Geneva, and simplified revised Geneva models), all models had moderate to good discriminative ability with AUC ranging from 0.75 (simplified revised Geneva score) to 0.80 (original and modified Wells rules)^47^. However, another study reported that the AUC of the Wells score and the revised Geneva score for predicting PTE risk in outpatients aged ≥ 65 years were 0.632 and 0.610, respectively^8^. A meta-analysis evaluating the Wells and revised Geneva scores for diagnosing PTE reported an AUC of 0.78 for the Wells score and 0.69 for the revised Geneva score^48^. Other clinical prediction rules for VTE have been developed, including the International Medical Prevention Registry on Venous Thromboembolism (IMPROVE) score^49^, Padua Prediction score (PPS)^50^, and Caprini score^51^. A study comparing the IMPROVE and IMPROVEDD scores, with the latter incorporating D-dimer values, found that the IMPROVEDD score had a higher AUC for VTE prediction at 42 days (0.621 vs. 0.588)^52^. The AUCs for the PPS ranged from 0.59 to 0.72^53–55^, although the score has not been extensively validated. Initially designed for both surgical and medical patients, the Caprini score has mainly been validated in surgical populations. Validation studies on the Caprini score in inpatients have shown AUCs ranging from 0.60 to 0.77^53–55^. The relatively low AUCs of the clinical prediction rules indicate an unmet need for improved VTE risk stratification.

Several retrospective studies have investigated ML-based models for predicting VTE, and have shown generally superior performance than that of clinical prediction rules^20–23,29,56^. Studies applying various ML algorithms to the same population have generally found that ensemble methods, particularly boosting methods, outperform non-ensemble methods^20,23,24^. Most previous studies focused on the inpatient population^20–23,56^ and used different definitions of reference standards. Often, the reference standard was confirmed using the ICD code for VTE and records of therapeutic treatment (e.g., thrombolytics or IVC filters), without verifying the diagnosis through adequate imaging examinations. Only a few studies have used imaging results as reference standards. One study examined the predictive ability of ML models in young-to middle-aged inpatients with VTE, validated by comprehensive diagnosis using various imaging methods (compression ultrasonography and contrast venography for DVT and pulmonary angiography, CT, and ventilation/perfusion scanning for PTE)^24^. This study reported an AUC of 0.875 using SVM. Another study that aimed to predict PTE that was confirmed using the gold standard method, CTPA, achieved an AUC of 0.799 using a gradient boosting decision tree^20^. To our knowledge, only one study has investigated the individualized risk of PTE in patients visiting the emergency department by using an ML model, and have reported an AUC of 0.89, using the elastic net^29^. That study relied on a comprehensive diagnosis utilizing various methods, including CTPA, ventilation/perfusion scanning, ultrasonography, angiography, and 90-day follow-up, to provide long-term risk predictions for patients visiting the emergency department. In comparison, our research focused on short-term PTE risk prediction for patients visiting the emergency department, up to 7 days before onset, with the reference standard validated by CTPA.

Our study had some limitations. First, the ML models were trained using retrospective data. Owing to the retrospective nature of the study, the types of available laboratory tests varied among patients, and the selection of laboratory tests was not standardized. We aimed to include as many test values as possible while minimizing the need to impute missing data. In addition, we were unable to compare the ML models with the Wells score and instead used the revised Geneva score, even though the Wells score has been reported to perform better for PTE risk assessment^48^. We did not apply the Wells score because it includes a clinical-judgment component (“PE more likely than an alternative diagnosis”) that cannot be reliably reconstructed from retrospective records. Second, this study was conducted at a single tertiary hospital. Given the number of predictors and the retrospective design, external validation in an independent cohort is necessary to assess model generalizability and potential overfitting. Third, the exclusion of patients who were not referred for CTPA may have introduced a referral bias by omitting patients with a low risk of PTE. Fourth, the main drawback of the PVI is its reliance on the random nature of permutations. This implies that different permutations yield slightly different results. However, the consistently large gap between the top-ranked variables (i.e., D-dimer level and aPTT) and the others across different ML models suggests that the inherent random variability of PVI does not significantly affect the interpretation of the most important variables. Fifth, the performance of our prediction model relies on the availability and quality of input data. Poor-quality predictor values can reduce accuracy and lead to unreliable predictions, potentially affecting clinical decision-making. Moreover, except for XGBoost, the machine learning models used in this study cannot handle missing predictor values, making them unsuitable for datasets with incomplete inputs. However, when complete data are available, these models can be applied without requiring advanced expertise in data preprocessing.

## Conclusion

Our findings suggest that various ML models can enhance the prediction of PTE risk compared with the revised Geneva score. The top-performing model, XGBoost demonstrated a highly discriminative probability score, which has the potential to significantly reduce the number of unnecessary CTPA scans in the emergency department. Nevertheless, in clinical practice, missed PTE can be fatal, necessitating a high sensitivity. Therefore, an appropriate sensitivity threshold should be carefully considered when applying ML models to rule out PTE.

## Supplementary Information

Below is the link to the electronic supplementary material.

Supplementary Material 1

Supplementary Material 2

Supplementary Material 3

Supplementary Material 4

## Data Availability

The anonymized datasets generated and analyzed during the current study are available from the corresponding author upon reasonable request.

## Code Availability

The full analysis code used in this study has been deposited in a public repository and is permanently accessible via Zenodo (10.5281/zenodo.17627734)^57^. This archived version corresponds exactly to the code used to generate all results reported in this manuscript. The repository is released under the MIT License. There are no access restrictions.

## Declarations

### Study protocol

A predefined formal protocol was not developed for this study. Full details of the methodology are provided in the main manuscript to ensure transparency and reproducibility.

### Study registration

This study was not registered. Relevant methods are transparently described within the manuscript.

### Patient and public involvement

There was no involvement of patients or the public in the design, conduct, reporting, interpretation, or dissemination of this study.
