# Supplementary material for: Machine learning improves prediction of pulmonary thromboembolism and reduces unnecessary computed tomography scans in the emergency department
Source: Sci Rep. 2026 Jan 9;16:4935. doi: 10.1038/s41598-025-34952-x (PMC12873316; doi:10.1038/s41598-025-34952-x)
Supplement: Supplementary file 1 — Supplementary Material 1 [file 41598_2025_34952_MOESM1_ESM.docx]

**Supplementary Table 1.** Characteristics of patients with and without PTE

| **Characteristic** | **Variable**  **Type** | **Overall**  **(n =** 2,525) | **PTE Negative**  **(n = 1,952)** | **PTE Positive**  **(n = 573)** | **p-value** |
| --- | --- | --- | --- | --- | --- |
| Sex | Binary |  |  |  | 0.007 |
| M |  | 1,214.0 (48.1%) | 967.0 (49.5%) | 247.0 (43.1%) |  |
| F |  | 1,311.0 (51.9%) | 985.0 (50.5%) | 326.0 (56.9%) |  |
| Age (years) | Continuous |  |  |  | 0.003 |
| Mean ± SD |  | 68.9 ± 16.2 | 69.4 ± 16.0 | 67.1 ± 16.7 |  |
| Median (IQR) |  | 73.0 (60.0, 80.0) | 73.5 (61.0, 81.0) | 71.0 (57.0, 79.0) |  |
| Pulse rate | Continuous |  |  |  | 0.368 |
| Mean ± SD |  | 96.8 ± 23.9 | 96.6 ± 24.4 | 97.5 ± 22.0 |  |
| Median (IQR) |  | 96.0 (80.0, 112.0) | 95.0 (79.0, 112.0) | 96.0 (82.0, 112.0) |  |
| N missing (% missing) |  | 19.0 (0.8%) | 15.0 (0.8%) | 4.0 (0.7%) |  |
| Respiratory rate | Continuous |  |  |  | 0.004 |
| Mean ± SD |  | 21.5 ± 6.1 | 21.7 ± 6.3 | 20.9 ± 5.3 |  |
| Median (IQR) |  | 20.0 (18.0, 24.0) | 20.0 (18.0, 24.0) | 20.0 (18.0, 22.0) |  |
| N missing (% missing) |  | 22.0 (0.9%) | 15.0 (0.8%) | 7.0 (1.2%) |  |
| Systolic blood pressure (mmHg) | Continuous |  |  |  | 0.178 |
| Mean ± SD |  | 131.0 ± 28.1 | 131.4 ± 29.0 | 129.7 ± 25.0 |  |
| Median (IQR) |  | 130.0 (113.0, 149.0) | 131.0 (113.0, 151.0) | 129.0 (114.0, 147.0) |  |
| N missing (% missing) |  | 21.0 (0.8%) | 15.0 (0.8%) | 6.0 (1.0%) |  |
| Diastolic blood pressure (mmHg) | Continuous |  |  |  | 0.216 |
| Mean ± SD |  | 73.5 ± 18.0 | 73.2 ± 18.2 | 74.3 ± 17.3 |  |
| Median (IQR) |  | 73.0 (62.0, 84.0) | 73.0 (62.0, 84.0) | 74.0 (63.0, 84.0) |  |
| N missing (% missing) |  | 22.0 (0.9%) | 17.0 (0.9%) | 5.0 (0.9%) |  |
| Body temperature (℃) | Continuous |  |  |  | 0.127 |
| Mean ± SD |  | 36.9 ± 0.8 | 36.9 ± 0.9 | 36.8 ± 0.7 |  |
| Median (IQR) |  | 36.8 (36.4, 37.2) | 36.8 (36.4, 37.2) | 36.8 (36.4, 37.2) |  |
| N missing (% missing) |  | 3.0 (0.1%) | 3.0 (0.2%) | 0.0 (0.0%) |  |
| Body mass index (kg/m^2^) | Continuous |  |  |  | <0.001 |
| Mean ± SD |  | 23.6 ± 4.4 | 23.3 ± 4.5 | 24.2 ± 4.2 |  |
| Median (IQR) |  | 23.3 (20.8, 26.1) | 23.1 (20.6, 25.9) | 23.9 (21.5, 26.6) |  |
| N missing (% missing) |  | 283.0 (11.2%) | 246.0 (12.6%) | 37.0 (6.5%) |  |
| D-dimer (μg/mL) | Continuous |  |  |  | <0.001 |
| Mean ± SD |  | 7.0 ± 7.4 | 6.1 ± 7.3 | 9.9 ± 7.0 |  |
| Median (IQR) |  | 3.8 (1.9, 10.5) | 3.3 (1.6, 8.3) | 8.2 (3.4, 17.8) |  |
| CK (IU/L) | Continuous |  |  |  | 0.705 |
| Mean ± SD |  | 262.8 ± 973.1 | 258.5 ± 938.1 | 277.8 ± 1,087.3 |  |
| Median (IQR) |  | 101.0 (81.1, 135.8) | 100.8 (80.4, 138.4) | 101.1 (83.1, 128.3) |  |
| N missing (% missing) |  | 55.0 (2.2%) | 33.0 (1.7%) | 22.0 (3.8%) |  |
| ANC (cells/µL) | Continuous |  |  |  | <0.001 |
| Mean ± SD |  | 9,949.5 ± 7,324.5 | 10,207.5 ± 7,768.2 | 9,071.0 ± 5,469.0 |  |
| Median (IQR) |  | 7,974.0 (5,215.0, 12,621.8) | 8,122.0 (5,181.5, 12,939.0) | 7,539.0 (5,278.0, 11,617.0) |  |
| N missing (% missing) |  | 1.0 (0.0%) | 1.0 (0.1%) | 0.0 (0.0%) |  |
| Segmented neutrophil (%) | Continuous |  |  |  | <0.001 |
| Mean ± SD |  | 78.5 ± 13.2 | 79.0 ± 13.3 | 76.9 ± 12.5 |  |
| Median (IQR) |  | 81.1 (69.8, 89.2) | 82.0 (70.4, 89.6) | 78.9 (67.9, 87.0) |  |
| N missing (% missing) |  | 1.0 (0.0%) | 1.0 (0.1%) | 0.0 (0.0%) |  |
| Eosinophil (%) | Continuous |  |  |  | 0.400 |
| Mean ± SD |  | 2.8 ± 3.6 | 2.8 ± 3.8 | 2.9 ± 3.1 |  |
| Median (IQR) |  | 2.0 (0.9, 3.6) | 1.9 (0.8, 3.5) | 2.2 (1.2, 3.9) |  |
| N missing (% missing) |  | 2.0 (0.1%) | 2.0 (0.1%) | 0.0 (0.0%) |  |
| Lymphocyte (%) | Continuous |  |  |  | <0.001 |
| Mean ± SD |  | 23.0 ± 12.5 | 22.1 ± 12.2 | 26.0 ± 13.2 |  |
| Median (IQR) |  | 20.9 (14.0, 29.7) | 20.0 (13.5, 28.7) | 23.9 (16.5, 33.7) |  |
| N missing (% missing) |  | 1.0 (0.0%) | 1.0 (0.1%) | 0.0 (0.0%) |  |
| WBC (10^3^/µL) | Continuous |  |  |  | <0.001 |
| Mean ± SD |  | 12.4 ± 7.8 | 12.7 ± 8.3 | 11.6 ± 5.7 |  |
| Median (IQR) |  | 10.4 (7.6, 15.2) | 10.4 (7.6, 15.5) | 10.0 (7.8, 14.1) |  |
| N missing (% missing) |  | 1.0 (0.0%) | 1.0 (0.1%) | 0.0 (0.0%) |  |
| RBC (10^6^/µL) | Continuous |  |  |  | <0.001 |
| Mean ± SD |  | 4.0 ± 0.7 | 4.0 ± 0.7 | 4.1 ± 0.6 |  |
| Median (IQR) |  | 4.0 (3.6, 4.4) | 4.0 (3.5, 4.4) | 4.1 (3.7, 4.6) |  |
| N missing (% missing) |  | 2.0 (0.1%) | 2.0 (0.1%) | 0.0 (0.0%) |  |
| Hemoglobin (g/dL) | Continuous |  |  |  | <0.001 |
| Mean ± SD |  | 12.3 ± 2.0 | 12.2 ± 2.0 | 12.6 ± 2.0 |  |
| Median (IQR) |  | 12.3 (10.9, 13.6) | 12.2 (10.8, 13.5) | 12.5 (11.1, 14.0) |  |
| N missing (% missing) |  | 1.0 (0.0%) | 1.0 (0.1%) | 0.0 (0.0%) |  |
| Hematocrit (%) | Continuous |  |  |  | <0.001 |
| Mean ± SD |  | 37.4 ± 5.8 | 37.2 ± 5.8 | 38.4 ± 5.6 |  |
| Median (IQR) |  | 37.2 (33.3, 41.3) | 36.9 (33.0, 40.8) | 38.2 (34.2, 42.5) |  |
| N missing (% missing) |  | 2.0 (0.1%) | 2.0 (0.1%) | 0.0 (0.0%) |  |
| PLT (10^3^/µL) | Continuous |  |  |  | 0.676 |
| Mean ± SD |  | 255.2 ± 116.5 | 254.7 ± 119.2 | 256.9 ± 107.0 |  |
| Median (IQR) |  | 236.0 (177.0, 306.0) | 235.0 (174.0, 305.5) | 239.0 (188.0, 308.0) |  |
| N missing (% missing) |  | 1.0 (0.0%) | 1.0 (0.1%) | 0.0 (0.0%) |  |
| Fibrinogen (mg/dL) | Continuous |  |  |  | <0.001 |
| Mean ± SD |  | 481.3 ± 181.6 | 493.5 ± 184.5 | 440.0 ± 164.9 |  |
| Median (IQR) |  | 455.0 (346.0, 589.3) | 468.0 (356.0, 609.0) | 409.0 (324.0, 533.0) |  |
| N missing (% missing) |  | 25.0 (1.0%) | 20.0 (1.0%) | 5.0 (0.9%) |  |
| aPTT (sec) | Continuous |  |  |  | <0.001 |
| Mean ± SD |  | 49.0 ± 30.4 | 45.4 ± 23.7 | 61.3 ± 44.4 |  |
| Median (IQR) |  | 39.8 (34.9, 47.9) | 39.1 (34.7, 46.4) | 42.3 (35.9, 58.2) |  |
| N missing (% missing) |  | 10.0 (0.4%) | 10.0 (0.5%) | 0.0 (0.0%) |  |
| PT INR (INR) | Continuous |  |  |  | 0.020 |
| Mean ± SD |  | 1.4 ± 1.0 | 1.3 ± 0.9 | 1.5 ± 1.2 |  |
| Median (IQR) |  | 1.2 (1.0, 1.3) | 1.1 (1.0, 1.3) | 1.2 (1.1, 1.5) |  |
| N missing (% missing) |  | 10.0 (0.4%) | 10.0 (0.5%) | 0.0 (0.0%) |  |
| Albumin (g/dL) | Continuous |  |  |  | 0.006 |
| Mean ± SD |  | 3.5 ± 0.6 | 3.5 ± 0.6 | 3.6 ± 0.6 |  |
| Median (IQR) |  | 3.6 (3.1, 4.0) | 3.6 (3.1, 4.0) | 3.6 (3.2, 4.0) |  |
| N missing (% missing) |  | 3.0 (0.1%) | 3.0 (0.2%) | 0.0 (0.0%) |  |
| Protein (g/dL) | Continuous |  |  |  | 0.180 |
| Mean ± SD |  | 6.6 ± 0.8 | 6.6 ± 0.8 | 6.7 ± 0.8 |  |
| Median (IQR) |  | 6.7 (6.1, 7.2) | 6.7 (6.1, 7.2) | 6.7 (6.2, 7.2) |  |
| N missing (% missing) |  | 3.0 (0.1%) | 3.0 (0.2%) | 0.0 (0.0%) |  |
| Cholesterol (mg/dL) | Continuous |  |  |  | <0.001 |
| Mean ± SD |  | 161.4 ± 47.6 | 158.2 ± 47.8 | 172.4 ± 45.3 |  |
| Median (IQR) |  | 158.0 (129.0, 190.0) | 155.0 (125.3, 186.0) | 171.0 (141.5, 199.5) |  |
| N missing (% missing) |  | 8.0 (0.3%) | 6.0 (0.3%) | 2.0 (0.3%) |  |
| CRP (mg/dL) | Continuous |  |  |  | <0.001 |
| Mean ± SD |  | 8.5 ± 8.6 | 8.9 ± 8.8 | 7.4 ± 7.8 |  |
| Median (IQR) |  | 5.5 (1.2, 13.7) | 6.0 (1.2, 14.3) | 4.5 (1.0, 12.3) |  |
| N missing (% missing) |  | 16.0 (0.6%) | 12.0 (0.6%) | 4.0 (0.7%) |  |
| Troponin I (ng/ml) | Continuous |  |  |  | 0.150 |
| Mean ± SD |  | 1.1 ± 9.3 | 0.9 ± 6.6 | 1.9 ± 15.3 |  |
| Median (IQR) |  | 0.0 (0.0, 0.1) | 0.0 (0.0, 0.1) | 0.0 (0.0, 0.2) |  |
| N missing (% missing) |  | 381.0 (15.1%) | 284.0 (14.5%) | 97.0 (16.9%) |  |
| HTN | Binary | 1,131.0 (44.8%) | 884.0 (45.3%) | 247.0 (43.1%) | 0.364 |
| DM | Binary | 627.0 (24.8%) | 507.0 (26.0%) | 120.0 (20.9%) | 0.015 |
| Previous VTE | Binary | 237.0 (9.4%) | 140.0 (7.2%) | 97.0 (16.9%) | <0.001 |
| Surgery or immobilization | Binary | 438.0 (17.3%) | 333.0 (17.1%) | 105.0 (18.3%) | 0.490 |
| Ischemic heart disease | Binary | 184.0 (7.3%) | 159.0 (8.1%) | 25.0 (4.4%) | 0.002 |
| Atrial fibrillation or atrial flutter | Binary | 243.0 (9.6%) | 204.0 (10.5%) | 39.0 (6.8%) | 0.010 |
| Stroke | Binary | 258.0 (10.2%) | 210.0 (10.8%) | 48.0 (8.4%) | 0.100 |
| Active cancer | Binary | 725.0 (28.7%) | 554.0 (28.4%) | 171.0 (29.8%) | 0.495 |
| Chemotherapy | Binary | 383.0 (15.2%) | 299.0 (15.3%) | 84.0 (14.7%) | 0.741 |
| Autoimmune disease | Binary | 27.0 (1.1%) | 23.0 (1.2%) | 4.0 (0.7%) | 0.487 |
| Coagulopathy | Binary | 11.0 (0.4%) | 3.0 (0.2%) | 8.0 (1.4%) | <0.001 |
| Anticoagulation | Binary | 412.0 (16.3%) | 320.0 (16.4%) | 92.0 (16.1%) | 0.898 |
| Unilateral leg pain | Binary | 316.0 (12.5%) | 191.0 (9.8%) | 125.0 (21.8%) | <0.001 |
| Hemoptysis | Binary | 4.0 (0.2%) | 4.0 (0.2%) | 0.0 (0.0%) | 0.580 |

Note: Unless otherwise specified, data in parentheses represent percentages. P-values were calculated using Fisher's exact test for dichotomous variables and Welch's Two-Sample t-test for continuous variables

CK, creatine kinase; ANC, absolute neutrophil count; WBC, white blood cell count; RBC, red blood cell count; PLT, platelet count; aPTT, activated partial thromboplastin time; PT INR, prothrombin time (international normalized ratio); CRP, C-reactive protein; HTN, hypertension; DM, diabetes; VTE, venous thromboembolism; PTE, pulmonary thromboembolism; DVT, deep venous thrombosis

**Supplementary Table 2.** Characteristics of the training and test datasets

| Characteristic | Overall  (n = 2,525) | Training dataset  (n = 2,025) | Test dataset  (n = 500) |
| --- | --- | --- | --- |
| PTE |  |  |  |
| Negative | 1,952.0 (77.3%) | 1,564.0 (77.2%) | 388.0 (77.6%) |
| Positive | 573.0 (22.7%) | 461.0 (22.8%) | 112.0 (22.4%) |
| Sex |  |  |  |
| M | 1,214.0 (48.1%) | 980.0 (48.4%) | 234.0 (46.8%) |
| F | 1,311.0 (51.9%) | 1,045.0 (51.6%) | 266.0 (53.2%) |
| Age (years) |  |  |  |
| Mean ± SD | 68.9 ± 16.2 | 68.7 ± 16.6 | 69.8 ± 14.4 |
| Median (IQR) | 73.0 (60.0, 80.0) | 73.0 (60.0, 80.0) | 73.0 (61.0, 80.0) |
| Pulse rate |  |  |  |
| Mean ± SD | 96.8 ± 23.8 | 96.5 ± 23.9 | 98.2 ± 23.5 |
| Median (IQR) | 96.0 (80.0, 112.0) | 95.0 (80.0, 112.0) | 97.5 (81.0, 113.0) |
| Respiratory rate |  |  |  |
| Mean ± SD | 21.5 ± 6.1 | 21.4 ± 5.7 | 22.2 ± 7.4 |
| Median (IQR) | 20.0 (18.0, 24.0) | 20.0 (18.0, 23.0) | 20.0 (18.0, 24.0) |
| Systolic blood pressure (mmHg) |  |  |  |
| Mean ± SD | 131.0 ± 28.0 | 130.8 ± 27.9 | 132.0 ± 28.4 |
| Median (IQR) | 130.0 (113.0, 149.0) | 130.5 (113.0, 149.0) | 130.0 (115.0, 152.0) |
| Diastolic blood pressure (mmHg) |  |  |  |
| Mean ± SD | 73.5 ± 18.0 | 73.4 ± 17.8 | 73.6 ± 18.5 |
| Median (IQR) | 73.0 (62.0, 84.0) | 73.0 (62.0, 84.0) | 73.0 (62.0, 83.0) |
| Body temperature (℃) |  |  |  |
| Mean ± SD | 36.9 ± 0.8 | 36.9 ± 0.9 | 36.8 ± 0.8 |
| Median (IQR) | 36.8 (36.4, 37.2) | 36.8 (36.4, 37.2) | 36.8 (36.4, 37.3) |
| Body mass index (kg/m^2^) |  |  |  |
| Mean ± SD | 23.6 ± 4.2 | 23.6 ± 4.0 | 23.6 ± 5.0 |
| Median (IQR) | 23.3 (21.1, 25.9) | 23.3 (21.1, 25.8) | 23.1 (20.6, 26.1) |
| D-dimer (µg/mL) |  |  |  |
| Mean ± SD | 7.0 ± 7.4 | 7.0 ± 7.6 | 7.1 ± 6.6 |
| Median (IQR) | 3.8 (1.9, 10.5) | 3.7 (1.9, 10.4) | 4.0 (1.9, 10.8) |
| CK (IU/L) |  |  |  |
| Mean ± SD | 259.3 ± 962.8 | 251.7 ± 884.4 | 290.2 ± 1,230.6 |
| Median (IQR) | 100.8 (81.7, 134.0) | 100.4 (81.0, 132.2) | 103.8 (83.1, 141.0) |
| ANC (cells/µL) |  |  |  |
| Mean ± SD | 9,947.8 ± 7,323.5 | 9,842.4 ± 7,334.5 | 10,375.0 ± 7,270.6 |
| Median (IQR) | 7,971.0 (5,216.0, 12,620.0) | 7,854.0 (5,129.0, 12,483.0) | 8,412.5 (5,426.0, 13,059.5) |
| Segmented neutrophil (%) |  |  |  |
| Mean ± SD | 78.5 ± 13.2 | 78.0 ± 13.4 | 80.3 ± 12.1 |
| Median (IQR) | 81.0 (69.8, 89.2) | 80.5 (69.4, 89.0) | 83.3 (71.9, 89.9) |
| Eosinophil (%) |  |  |  |
| Mean ± SD | 2.8 ± 3.6 | 2.7 ± 3.8 | 3.0 ± 3.0 |
| Median (IQR) | 2.0 (0.9, 3.6) | 1.9 (0.9, 3.5) | 2.3 (1.0, 4.0) |
| Lymphocyte (%) |  |  |  |
| Mean ± SD | 23.0 ± 12.5 | 23.1 ± 12.7 | 22.7 ± 11.7 |
| Median (IQR) | 20.9 (14.0, 29.7) | 20.7 (14.1, 29.8) | 21.6 (13.5, 29.6) |
| WBC (10^3^/µL) |  |  |  |
| Mean ± SD | 12.4 ± 7.8 | 12.4 ± 7.8 | 12.7 ± 7.5 |
| Median (IQR) | 10.4 (7.6, 15.2) | 10.2 (7.6, 15.2) | 10.8 (7.8, 15.4) |
| RBC (10^6^/µL) |  |  |  |
| Mean ± SD | 4.0 ± 0.7 | 4.0 ± 0.7 | 4.0 ± 0.7 |
| Median (IQR) | 4.0 (3.6, 4.4) | 4.0 (3.6, 4.4) | 4.0 (3.6, 4.4) |
| Hemoglobin (g/dL) |  |  |  |
| Mean ± SD | 12.3 ± 2.0 | 12.4 ± 2.0 | 12.2 ± 1.9 |
| Median (IQR) | 12.3 (10.9, 13.6) | 12.3 (10.9, 13.7) | 12.2 (10.8, 13.5) |
| Hematocrit (%) |  |  |  |
| Mean ± SD | 37.4 ± 5.8 | 37.5 ± 5.8 | 37.3 ± 5.7 |
| Median (IQR) | 37.2 (33.3, 41.3) | 37.2 (33.3, 41.3) | 36.9 (33.2, 41.2) |
| PLT (10^3^/µL) |  |  |  |
| Mean ± SD | 255.2 ± 116.5 | 255.0 ± 116.2 | 256.1 ± 117.6 |
| Median (IQR) | 236.0 (177.0, 306.0) | 236.0 (177.0, 306.0) | 238.0 (177.0, 310.0) |
| Fibrinogen (mg/dL) |  |  |  |
| Mean ± SD | 481.0 ± 180.9 | 477.4 ± 180.2 | 495.9 ± 183.3 |
| Median (IQR) | 455.0 (347.0, 589.0) | 451.0 (344.0, 585.0) | 469.0 (367.5, 603.5) |
| aPTT (sec) |  |  |  |
| Mean ± SD | 49.0 ± 30.4 | 49.0 ± 30.9 | 48.8 ± 28.4 |
| Median (IQR) | 39.8 (34.9, 47.8) | 39.6 (34.7, 47.7) | 40.4 (35.4, 48.9) |
| PT INR (INR) |  |  |  |
| Mean ± SD | 1.4 ± 1.0 | 1.4 ± 1.0 | 1.4 ± 0.7 |
| Median (IQR) | 1.2 (1.0, 1.3) | 1.1 (1.0, 1.3) | 1.2 (1.1, 1.4) |
| Albumin (g/dL) |  |  |  |
| Mean ± SD | 3.5 ± 0.6 | 3.6 ± 0.6 | 3.5 ± 0.6 |
| Median (IQR) | 3.6 (3.1, 4.0) | 3.6 (3.2, 4.0) | 3.5 (3.1, 3.9) |
| Protein (g/dL) |  |  |  |
| Mean ± SD | 6.6 ± 0.8 | 6.6 ± 0.8 | 6.6 ± 0.8 |
| Median (IQR) | 6.7 (6.1, 7.2) | 6.7 (6.2, 7.2) | 6.6 (6.1, 7.1) |
| Cholesterol (mg/dL) |  |  |  |
| Mean ± SD | 161.4 ± 47.5 | 161.9 ± 46.9 | 159.5 ± 50.1 |
| Median (IQR) | 158.0 (129.0, 190.0) | 158.0 (130.0, 189.0) | 155.0 (123.0, 193.0) |
| CRP (mg/dL) |  |  |  |
| Mean ± SD | 8.5 ± 8.6 | 8.4 ± 8.6 | 9.0 ± 8.6 |
| Median (IQR) | 5.5 (1.2, 13.7) | 5.2 (1.1, 13.5) | 6.7 (1.5, 14.2) |
| Troponin I (ng/ml) |  |  |  |
| Mean ± SD | 1.0 ± 8.6 | 1.0 ± 9.4 | 0.6 ± 3.9 |
| Median (IQR) | 0.0 (0.0, 0.1) | 0.0 (0.0, 0.1) | 0.0 (0.0, 0.1) |
| HTN | 1,131.0 (44.8%) | 887.0 (43.8%) | 244.0 (48.8%) |
| DM | 627.0 (24.8%) | 501.0 (24.7%) | 126.0 (25.2%) |
| Previous VTE | 237.0 (9.4%) | 188.0 (9.3%) | 49.0 (9.8%) |
| Surgery or immobilization | 438.0 (17.3%) | 351.0 (17.3%) | 87.0 (17.4%) |
| Ischemic heart disease | 184.0 (7.3%) | 144.0 (7.1%) | 40.0 (8.0%) |
| Atrial fibrillation or atrial flutter | 243.0 (9.6%) | 187.0 (9.2%) | 56.0 (11.2%) |
| Stroke | 258.0 (10.2%) | 204.0 (10.1%) | 54.0 (10.8%) |
| Active cancer | 725.0 (28.7%) | 566.0 (28.0%) | 159.0 (31.8%) |
| Chemotherapy | 383.0 (15.2%) | 292.0 (14.4%) | 91.0 (18.2%) |
| Autoimmune disease | 27.0 (1.1%) | 22.0 (1.1%) | 5.0 (1.0%) |
| Coagulopathy | 11.0 (0.4%) | 10.0 (0.5%) | 1.0 (0.2%) |
| Anticoagulation | 412.0 (16.3%) | 327.0 (16.1%) | 85.0 (17.0%) |
| Unilateral leg pain | 316.0 (12.5%) | 269.0 (13.3%) | 47.0 (9.4%) |
| Hemoptysis | 4.0 (0.2%) | 4.0 (0.2%) | 0.0 (0.0%) |

CK, creatine kinase; ANC, absolute neutrophil count; WBC, white blood cell count; RBC, red blood cell count; PLT, platelet count; aPTT, activated partial thromboplastin time; PT INR, prothrombin time (international normalized ratio); CRP, C-reactive protein; HTN, hypertension; DM, diabetes; VTE, venous thromboembolism; PTE, pulmonary thromboembolism; DVT, deep venous thrombosis

**Supplementary Table 3.** Specifications of the machine-learning models

| Model | Package | Hyperparameter | Value | Definition |
| --- | --- | --- | --- | --- |
| Boosted tree (XGBoost) | XGBoost | nrounds | 220 | The number of trees to be added to the model. |
|  |  | eta | 0.02 | The step size at each boosting iteration (learning rate) |
|  |  | max_depth | 8 | The maximum depth of each decision tree |
|  |  | colsample_bytree | 0.1 | The fraction of variables to be randomly sampled for each tree |
|  |  | early_stopping_rounds | 50 | The number of rounds (iterations) with no improvement in the evaluation metric after which training will be stopped. |
| Random Forest | ranger | mtry | 5 | The fraction of variables to be randomly sampled for each tree |
|  |  | num.trees | 310 | The number of trees to be added to the model. |
|  |  | min.node.size | 5 | The minimum number of data points in a node that are required for the node to be split further. |
| Elastic net regression | glmnet | lambda | 0.01 | The regularization strength for models |
|  |  | alpha | 0.1 | The mixing ratio between Lasso (L1) and Ridge (L2) regularization in the Elastic Net model. When alpha is set to 1, the model behaves like a lasso regression (pure L1 penalty). When alpha is set to 0, the model behaves like a ridge regression (pure L2 penalty). |
| SVM (Linear kernel) | kernlab | C | 0.003 | The cost of predicting a sample within or on the wrong side of the margin |
| SVM (Radial kernel) | kernlab | C | 0.1 | The cost of predicting a sample within or on the wrong side of the margin |
|  |  | sigma | 0.03 | The width of the Gaussian function and determines the extent of influence that a single training example has. |
| Feed-forward neural network | nnet | size | 5 | The number of units in the hidden layer |
|  |  | maxit | 10 | The number of training iterations. |
|  |  | decay | 0.001 | The amount of weight decay. |

PTE, pulmonary thromboembolism; SVM, support vector machine;


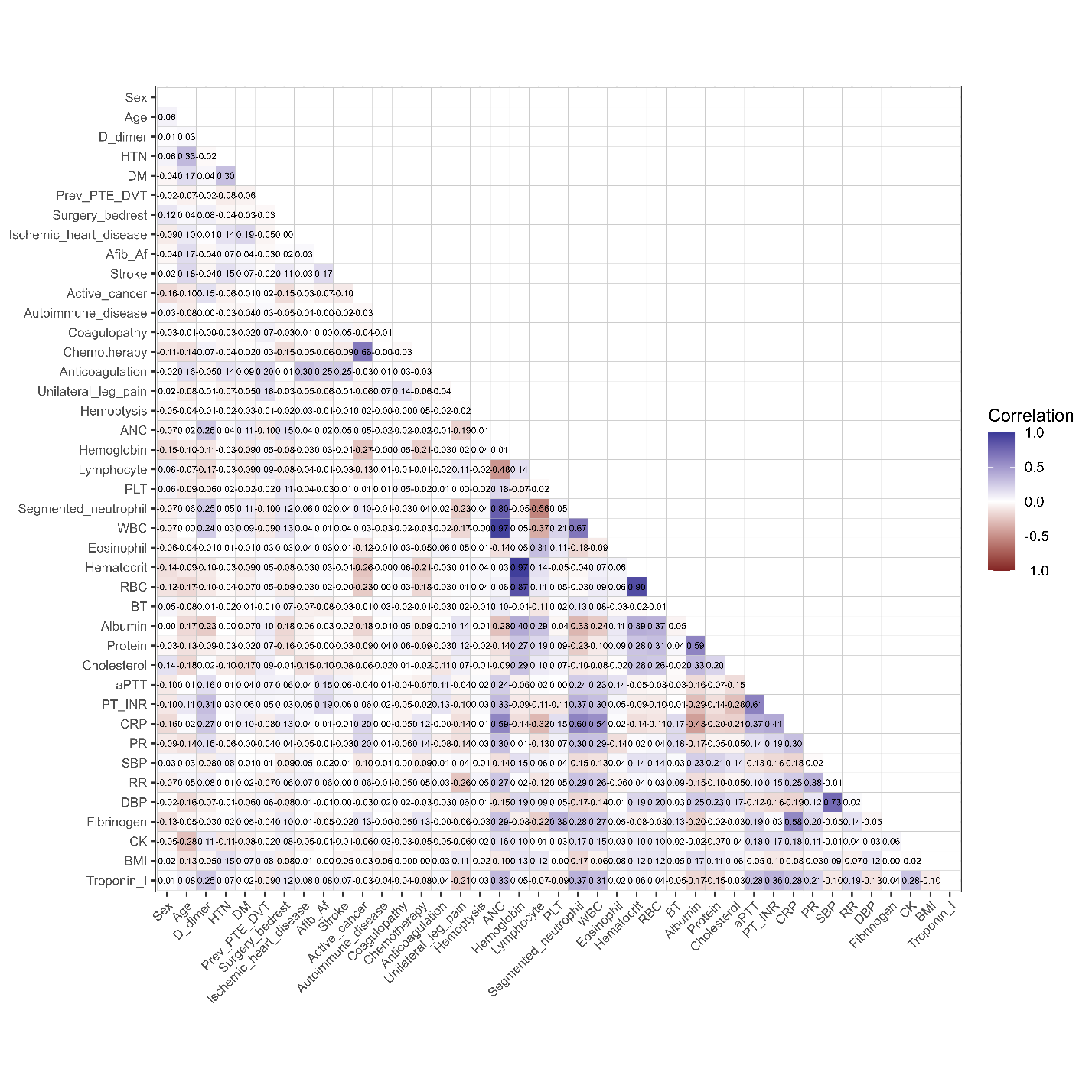


**Supplementary Figure 1.** Pairwise correlation coefficient between predictors

Abbreviations: Afib_Af, atrial fibrillation or atrial flutter; ANC, absolute neutrophil count; aPTT, activated partial thromboplastin time; BMI, body mass index; BT, body temperature; CK, creatine kinase; CRP, C-reactive protein; DBP, diastolic blood pressure; PLT, platelet count; PR, pulse rate; Prev_PTE_DVT, previous pulmonary thromboembolism or deep vein thrombosis; PT_INR, prothrombin time international normalized ratio; RBC, red blood cell count; RR, respiratory rate; SBP, systolic blood pressure; WBC, white blood cell count.


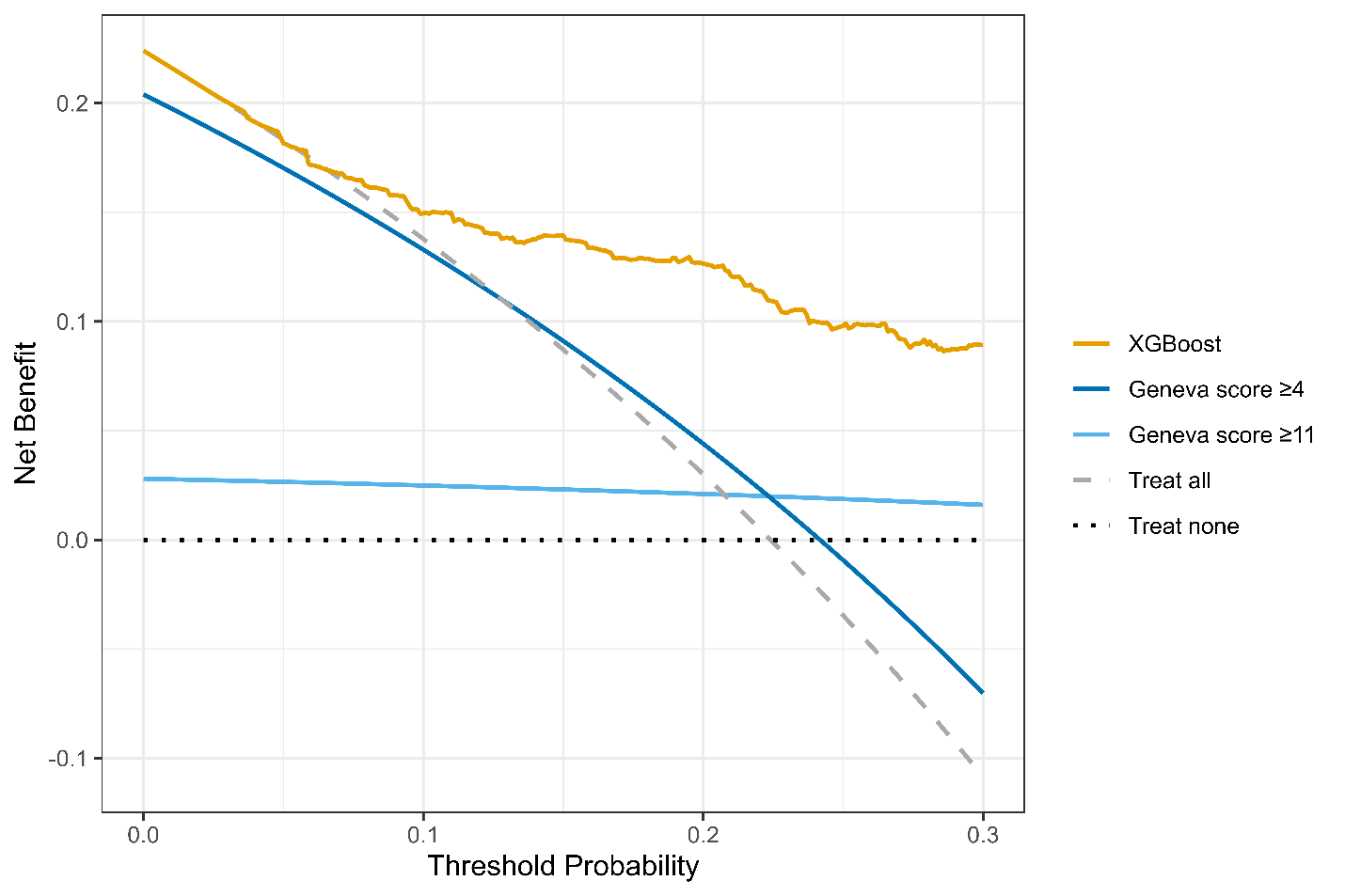


**Supplementary Figure 2.** Decision curve analysis of the XGBoost model

This analysis compares the XGBoost model with the established revised Geneva score thresholds at 4 points (low vs. intermediate/high) and 11 points (intermediate vs. high).


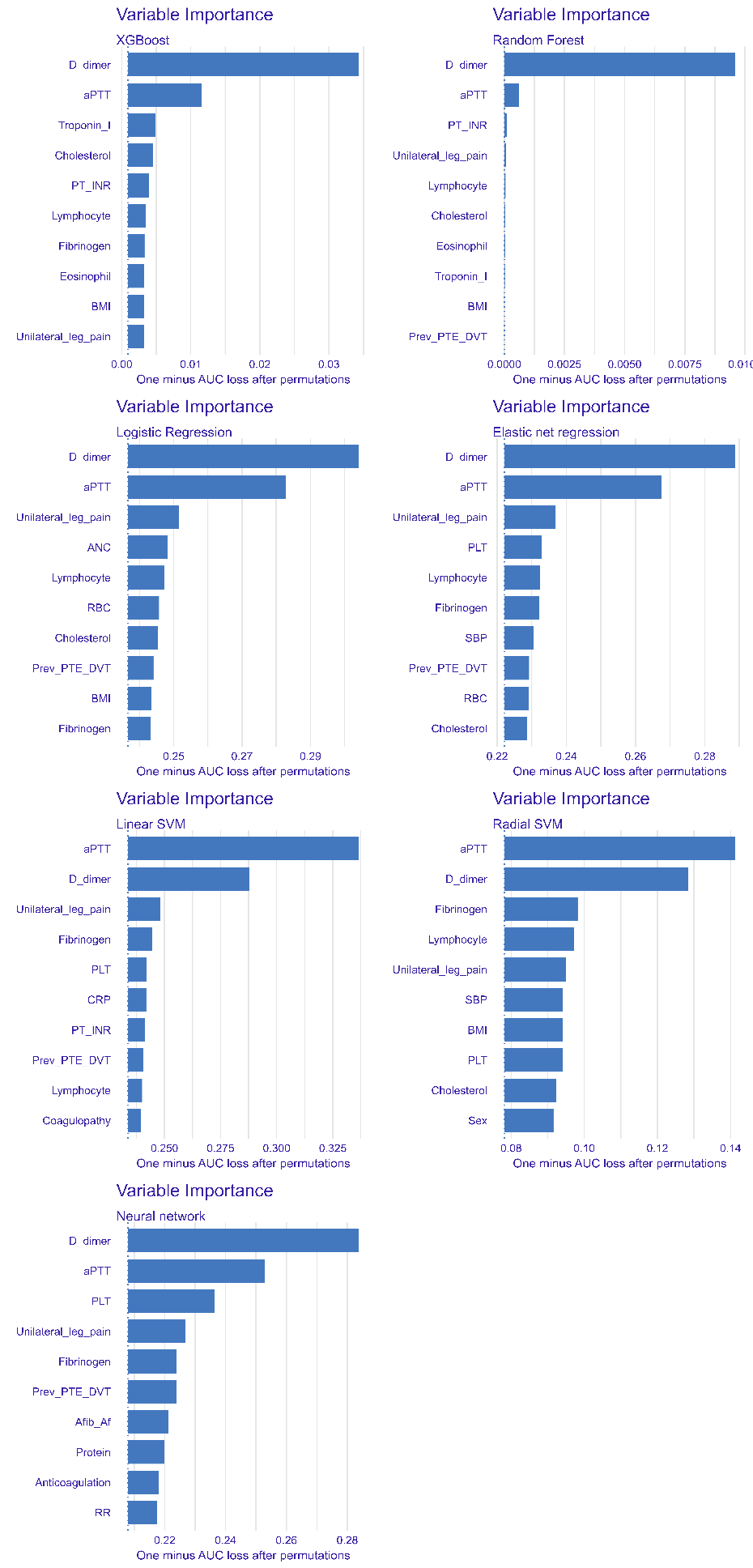


**Supplementary Figure 3.** Variable importance plots of the machine-learning models

Abbreviations: aPTT, activated partial thromboplastin time; Afib_Af, atrial fibrillation or atrial flutter; ANC, absolute neutrophil count; BMI, body mass index; CRP, C-reactive protein; PLT, platelet count; Prev_PTE_DVT, previous pulmonary thromboembolism or deep vein thrombosis; PT_INR, prothrombin time (international normalized ratio); RBC, red blood cell count; RR, respiratory rate; SBP, systolic blood pressure.
